# Selecting EEG channels and features using multi-objective optimization for accurate MCI detection: validation using leave-one-subject-out strategy

**DOI:** 10.1038/s41598-024-63180-y

**Published:** 2024-05-30

**Authors:** Majid Aljalal, Saeed A. Aldosari, Marta Molinas, Fahd A. Alturki

**Affiliations:** 1 Department of Electrical Engineering, College of Engineering, King Saud University, Riyadh, Saudi Arabia; 2 Department of Engineering Cybernetics, Norwegian University of Science and Technology, Trondheim, Norway

**Keywords:** MCI, EEG channel selection, Feature selection, Machine learning, Multi-objective optimization, NSGA, Diagnostic markers, Neurological disorders, Biomedical engineering, Learning algorithms

## Abstract

Effective management of dementia requires the timely detection of mild cognitive impairment (MCI). This paper introduces a multi-objective optimization approach for selecting EEG channels (and features) for the purpose of detecting MCI. Firstly, each EEG signal from each channel is decomposed into subbands using either variational mode decomposition (VMD) or discrete wavelet transform (DWT). A feature is then extracted from each subband using one of the following measures: standard deviation, interquartile range, band power, Teager energy, Katz's and Higuchi's fractal dimensions, Shannon entropy, sure entropy, or threshold entropy. Different machine learning techniques are used to classify the features of MCI cases from those of healthy controls. The classifier's performance is validated using leave-one-subject-out (LOSO) cross-validation (CV). The non-dominated sorting genetic algorithm (NSGA)-II is designed with the aim of minimizing the number of EEG channels (or features) and maximizing classification accuracy. The performance is evaluated using a publicly available online dataset containing EEGs from 19 channels recorded from 24 participants. The results demonstrate a significant improvement in performance when utilizing the NSGA-II algorithm. By selecting only a few appropriate EEG channels, the LOSO CV-based results show a significant improvement compared to using all 19 channels. Additionally, the outcomes indicate that accuracy can be further improved by selecting suitable features from different channels. For instance, by combining VMD and Teager energy, the SVM accuracy obtained using all channels is 74.24%. Interestingly, when only five channels are selected using NSGA-II, the accuracy increases to 91.56%. The accuracy is further improved to 95.28% when using only 8 features selected from 7 channels. This demonstrates that by choosing informative features or channels while excluding noisy or irrelevant information, the impact of noise is reduced, resulting in improved accuracy. These promising findings indicate that, with a limited number of channels and features, accurate diagnosis of MCI is achievable, which opens the door for its application in clinical practice.

## Introduction

Dementia progressively impairs cognitive functions, including memory, speech, and thinking skills, significantly impacting daily life^1^. This ailment is more frequently seen in those aged 60 and older. The challenge in remembering recent events is the earliest and most distinct signs of dementia^2^. Mild cognitive impairment (MCI) is considered the early phase of several types of dementia, including Alzheimer’s disease (AD). It, namely MCI, is marked by noticeable yet not overly disruptive cognitive changes for individuals and their families^3,4^. MCI doesn't fully align with the standard for diagnosing dementia, or AD, due to its milder effect on everyday activities. However, it places individuals at a considerable risk of developing dementia, for instance, with about 15–20% of MCI individuals advancing to AD annually^5^. Despite the recent FDA approval of Lecanemab, a novel medication for AD treatment^6^, the early detection of AD during the MCI stage assumes significant importance in impeding disease progression and promoting the advancement of non-pharmacological therapies. Usually, diagnosing MCI or AD requires a lengthy and multi-step procedure involving a Mini-Mental State Examination (MMSE), blood tests, neurological exams, and spinal fluid analysis. Therefore, it is essential to develop safe, objective, and practical methods for early MCI detection to prompt timely intervention.

Numerous research efforts have been dedicated to exploring various techniques for diagnosing MCI, including approaches based on magnetic resonance imaging (MRI)^7,8^, positron emission tomography (PET)^9^, computed tomography (CT)^10^, and combined methods^11^. Electroencephalography (EEG) has also gained prominence as a non-invasive modality for the automated diagnosis of brain disorders. This technique involves the placement of electrodes on the scalp to monitor the electrical activity generated by brain neurons^12^. The detail with which the EEG can map brain activity is contingent upon the electrode number and arrangement. Offering advantages like high temporal resolution, portability, affordability, and operational efficiency, EEG stands out from other imaging methods such as MRI, CT, and PET^12^. EEG, in combination with machine learning techniques, has been increasingly used to categorize a variety of neurological disorders. It has proven effective in diagnosing conditions like AD^13,14^, autism spectrum disorder (ASD)^15,16^, major depressive disorder^17^, epilepsy^16,18^, schizophrenia^19^, and Parkinson’s disease (PD)^20,21^, and it has also been applied in tasks such as emotion recognition^22^. Recent studies have leveraged EEG signals and machine learning to automate the detection of MCI. These investigations have delved into distinct EEG paradigms for tasks and resting states^23–40^. Task-state EEG requires individuals to engage in activities, such as responding to sequential speech sounds, during the recording^23^. Conversely, resting-state EEG tracks brain activity in a more passive state, either with the eyes closed or open, without any task involvement. This method of EEG data collection is especially advantageous for elderly subjects, as it is less demanding, more reflective of everyday conditions, and generally more comfortable.

A wide range of research (cited in references^24–35^) has been conducted on the use of resting-state EEGs for detecting MCI. These studies have utilized various techniques to create biomarkers for MCI identification and employed different classifiers to distinguish between MCI patients and healthy controls (HC). For example, Kashefpoor et al.^24^ applied several spectral analyses to EEG data from 11 MCI patients and 16 HCs. They used a neuro-fuzzy method combined with a K-nearest neighbor (KNN) technique, achieving 88.89% accuracy in feature classification. This group^25^ also developed a supervised dictionary learning method, named CLC-KSVD, for EEG analysis, attaining 88.9% accuracy in classification using the left-temporal area, which contains the channels F7, T3, and T5. Using the dataset from^24^, Hadiyoso et al.^26^ employed KNN for classifying power spectral features, obtaining 81.5% accuracy. Yin et al.^27^ balanced the MCI and HC sample sizes from the same dataset, applying stationary wavelet transformation (SWT) for signal enhancement and using statistical measures for feature extraction. Their support vector machine (SVM) classifier reached 96.94% accuracy. Siuly et al.^28^ analyzed the^24^ dataset with auto-regressive and permutation entropy methods, achieving 98.78% accuracy using an extreme learning machine classifier. Hsiao et al.^29^ introduced features that were all based on relative power and utilized Fisher's method for feature selection. They attained 90.20% accuracy using a SVM classifier on a 30-channel EEG dataset of 27 HC and 24 MCI participants. Alvi et al.^30^ explored deep learning approaches using several Long Short-Term Memory (LSTM) models, identifying the most effective model with 96.41% accuracy. Lee et al.^31^ retrieved a wide range of features, including power spectral density and complexity measures, from their dataset, achieving up to 86.85% accuracy with the SVM classifier. Movahed et al.^32^ extracted spectral and nonlinear biomarkers from EEG data that contains 16 HC and 18 MCI individuals, achieving 99.4% accuracy with a linear SVM classifier. Said and Göker^33^ applied the discrete wavelet transform (DWT) leader for feature extraction, attaining 93.50% accuracy with the AdaBoostM1 algorithm. Aljalal et al.^34^ used empirical mode decomposition (EMD) to decompose EEG data, achieving 97.60% accuracy with a KNN classifier. These studies collectively highlight the evolving and diverse approaches to EEG-based MCI detection. Lastly, Ahad et al.^35^ employed the^24^ dataset and explored the convolutional neural network (CNN) deep learning model, achieving an accuracy of 84.28% with the LOSO.

Several studies have focused on differentiating MCI, AD, and HC. For example, Fiscon et al.^36^ analyzed data from 109 subjects, including 37 with MCI, 49 with AD, and 23 HCs, assessing the utility of Fourier and wavelet transforms in this context. They discovered that the combining of DWT and a Decision Tree (DT) classifier achieves an 83.3% accuracy in differentiating MCI from HC using holdout validation and a 93.3% accuracy using tenfold cross-validation. In another study, Sharma et al.^37^ used the SVM classifier to analyze EEG data from 44 subjects, comprising 16 with MCI, 15 with dementia, and 13 HCs, across various conditions such as eye-open and eye-close states, finger-tapping, and continuous performance tests. They employed eight different measures, including power spectral density (PSD) and various spectral and fractal features. For MCI versus HC, the accuracy reached 84.1%. in the resting state with eyes open. Oltu et al.^38^ worked with EEG data containing 11 MCI, 8 AD, and 11 HC subjects, applying DWT, power spectral density, and interhemispheric coherence measures. A bagged tree classifier led to an accuracy of 96.50% in their classification tasks. More recently, Pirrone et al.^39^ examined EEG signals from 105 individuals, including 37 with MCI, 48 with AD, and 20 HCs. They focused on the power intensity in both high and low frequency bands and used SVM, DT, and K-nearest neighbors (KNN) classifiers for various classification scenarios. In distinguishing MCI from HC, the KNN classifier achieved 95% accuracy.

With the exception of studies^27,29,31,32,37^, contemporary research in MCI classification has largely concentrated on enhancing accuracy through feature extraction, often overlooking the significance of channel and feature selection. Efficiently selecting channels or specific features from channel subsets not only boosts classification precision but also aids in creating user-friendly and portable MCI detection systems. Regarding channel selection, the authors of^27^ attempted to lower the number of channels using an incremental evaluation method but couldn't identify a subset surpassing the accuracy achieved with the complete set of 19 channels. They found that accuracy improved with an increasing number of channels, yet the peak accuracy of 96.94% was only attained when all channels were utilized. The researchers in^31^ assessed classification accuracies with subsets of channels in symmetrical combinations (two, four, six, and eight electrodes). For example, in two-channel assessments, specific channel pairs like Fp1 with Fp2 and F7 with F8 were selected. This approach, however, did not consider numerous other two-channel combinations that might yield higher accuracies. The same limitation applied to larger channel combinations. The choice of symmetric channel pairs was driven by the challenge of manually exploring all possible combinations. Regarding selecting features belonging to a number of channels, in the study^29^, Fisher’s class separability criterion was applied to identify the most effective channels and frequency subbands for extracting key features. This approach led to seven features from five different channels, resulting in the highest classification accuracy of 90.25%. In^32^, 431 features were aggregated from all channels using various measures. For feature selection, a backward-elimination method was used to select 361 features, leading to an accuracy of 91.1%. The study^37^ used an ANOVA test to select the best measures. For distinguishing MCI from HC, power spectral density, spectral entropy, spectral kurtosis, and fractional dimension measures were selected. Although these studies have made significant strides, there remains a need for more systematic, generalizable, and effective methods in channel and feature selection.

Besides, there is a noticeable gap in most of the previous studies^27,28,30,32–34,36–39^, as the methods proposed in these studies were evaluated using intra-subject validation methods, such as k-fold cross-validation (CV). Intra-subject methods have a drawback in that they can potentially introduce a classification bias due to data leakage. Data leakage occurs when information from a specific subject is inadvertently included in both the training and testing phases, which can lead to biased results. To mitigate this issue, employing inter-subject classification methods like leave-one-subject-out (LOSO) for validation becomes crucial. LOSO mimics real-world scenarios and helps prevent data leakage, ensuring more reliable and unbiased evaluations. Hold-out cross-validation (which is used in^24–26,36^) has a similar advantage, but the difference is that the LOSO CV ensures that all subjects have been used for testing through a number of iterations equal to the number of subjects. Therefore, LOSO is considered to be more generalized than the holdout technique. Of all the previous studies, only^29,35^ used LOSO for validation. The study^29^ also explored selecting channels using Fisher’s class separability criterion, reporting an accuracy of 90.25% on a non-public dataset. The study^35^ didn’t develop a feature extraction method but manually explored frequency band-based features using a CNN model.

Thus, there remains a necessity to introduce alternative and more effective approaches for selecting channels or features that have the potential to improve the classification accuracy. Additionally, it is crucial to evaluate the accuracy using an inter-subject classification-based method. In a previous study^40^, we examined the potential of decreasing the quantity of EEG channels while simultaneously preserving the accuracy of classification. For this aim, various strategies, including optimization techniques and greedy algorithms, were employed. The results presented in^40^ indicated that optimization techniques exhibited superior automatic channel selection capabilities compared to the greedy methods. However, like other studies^40^, relied on the k-fold CV for validation. In this study, we aim to address these limitations by exploring EEG channel (and feature) selection using multi-objective optimization while evaluating classification accuracy using LOSO CV. Our goal is to develop a precise MCI detection system with a minimal number of electrodes. This study contributes in numerous ways, including the following:

- Conducting investigations on variational mode decomposition (VMD) and DWT methods for decomposing EEG signals, followed by the extraction of various non-linear, spectral, and functional connectivity features to develop appropriate biomarkers for detecting MCI.
- Utilization of the multi-objective optimization method, the non-dominated sorting genetic algorithm (NSGA-II), for EEG channel selection aims to minimize the number of required EEG channels while simultaneously improving classification accuracy.
- Exploration of feature selection using NSGA-II to further enhance the accuracy of MCI identification. To the best of our knowledge, our group is the first to employ a heuristic optimization method for the purpose of selecting channels and features in MCI identification.
- Application of NSGA-II for selecting optimal parameters for classifiers and investigating different machine learning techniques.
- Validation of classification performance using LOSO CV, which involves inter-subject classification.
- Evaluation of the suggested approaches on a publicly available dataset previously used in related studies^24–28,30,35^.

“Materials and methods” section describes the EEG data utilized in the study in detail, as well as the procedures used for EEG signal processing and channel/feature selection. “Results” section includes the study's findings. “Discussion” section includes the discussion and the comparison with the studies in the literature. "Conclusion and future work" section wraps up the paper and makes suggestions for further study.

## Materials and methods

**Figure 1:**
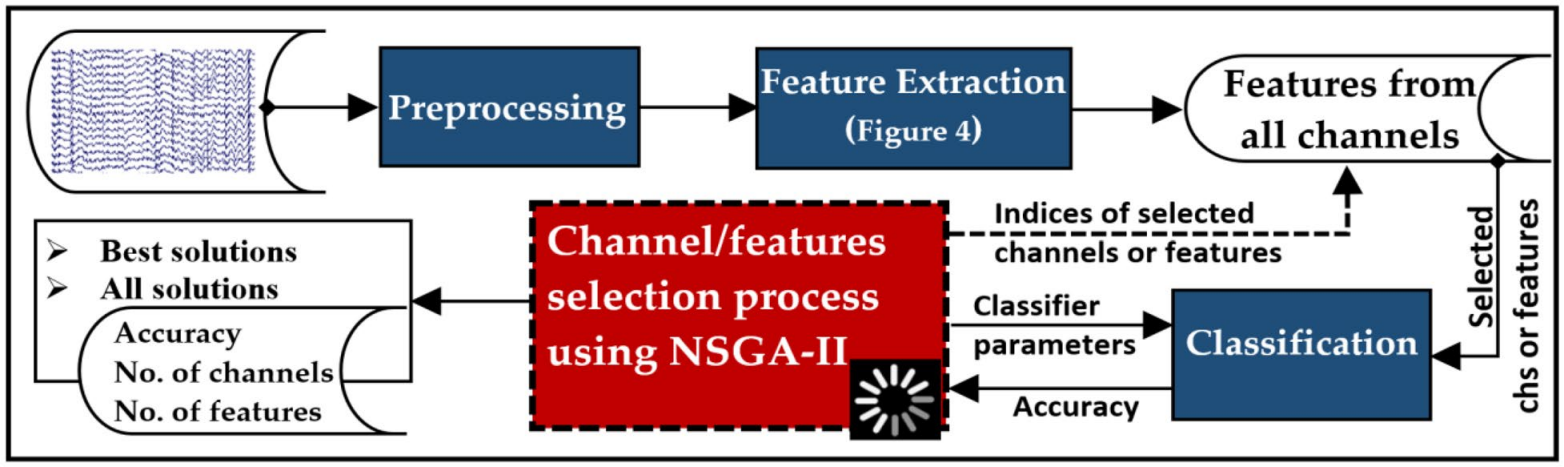
A high-level overview of the EEG signal processing stages followed in this study.

Figure 1 presents a concise summary of the main stages involved in EEG data processing and channel (and feature) selection. The initial stage focuses on reading and preprocessing the raw EEG signals to remove artifacts and concentrate on the desired frequency band. Additionally, the cleaned signals are also segmented into non-overlapping multi-channel segments. In the feature extraction, each signal within a segment is decomposed using either DWT or VMD, resulting in sub-signals that exhibit distinct frequency bands (for more detailed information, refer to Fig. 4). From each sub-signal, a single feature value is extracted by applying one of the proposed measures (details in “Feature extraction (FE)” Section). The values obtained from all signals within a segment are aggregated to create a feature vector. This process is then repeated for the remaining segments, resulting in the construction of other feature vectors. For selecting channels or features, the NSGA-II algorithm employs a dynamic approach to select channels or features based on predefined objectives, maximizing classification accuracy while simultaneously decreasing the quantity of channels or features. The algorithm returns the indices of the selected channels or features, as illustrated in Fig. 1. When it comes to channel selection, all features associated with the selected channels are utilized during classification. Conversely, in the feature selection stage, only the selected features are utilized. Each stage is further elaborated upon in the subsequent subsections, providing more comprehensive details.

### Subjects and data pre-processing

**Figure 2:**
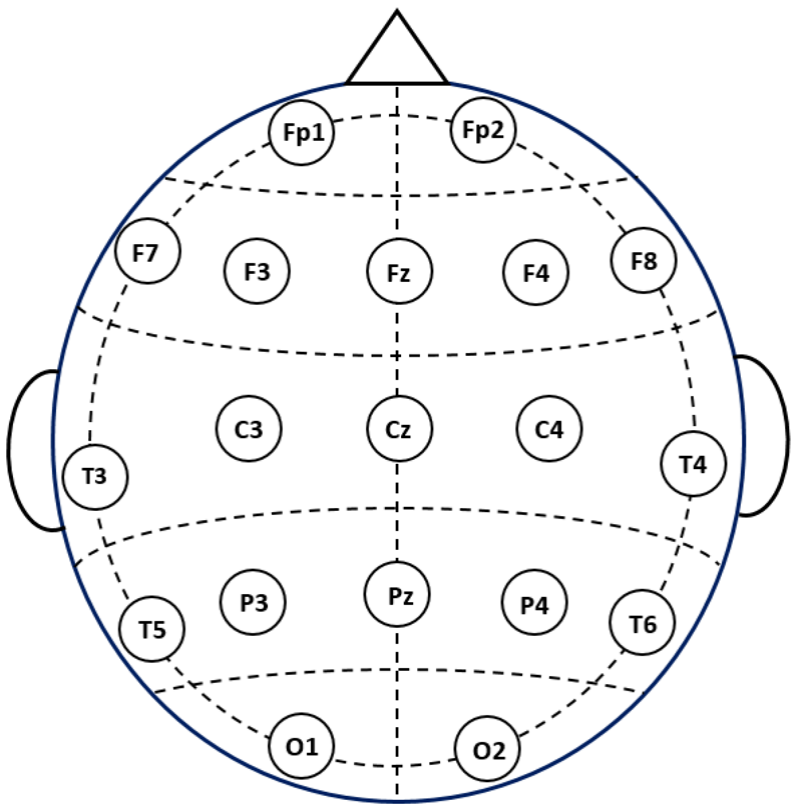
Placement of the 19 EEG channels used from^40^.

An open dataset, which can be found in^41^, is utilized. The dataset consists of 11 patients with MCI and 16 healthy individual cases (HC). Every participant had at least completed their primary schooling. EEG recordings, participant recruitment, cognitive assessments, and other procedures were conducted in Isfahan, Iran, at Noor Hospital. Prospective participants with a history of dementia, significant physical ailments, substance abuse, brain injuries, or severe mental disorders were excluded from the study. When a subject's MMSE score is between 21 and 26, he is regarded as having MCI, whereas scores above 27 are considered normal. For the HC and MCI groups, the mean and standard deviation of the subjects' ages are 63.84.3 and 65.74.9, respectively. All subjects recorded their EEGs in the morning while lying down with their eyes closed in a quiet environment. For positioning 19 EEG channels, the 10–20 International System was adopted^42^. Figure 2 illustrates the specific locations of these 19 channels. At a sampling frequency of 256 Hz, EEG data was recorded using a 32-channel digital EEG apparatus for a period of 30 min. For more information on recording details, we refer the reader to^24,42^. Due to the potential volunteer fatigue brought on by extended recording, this study only considered the first 10 min of data.

**Figure 3:**
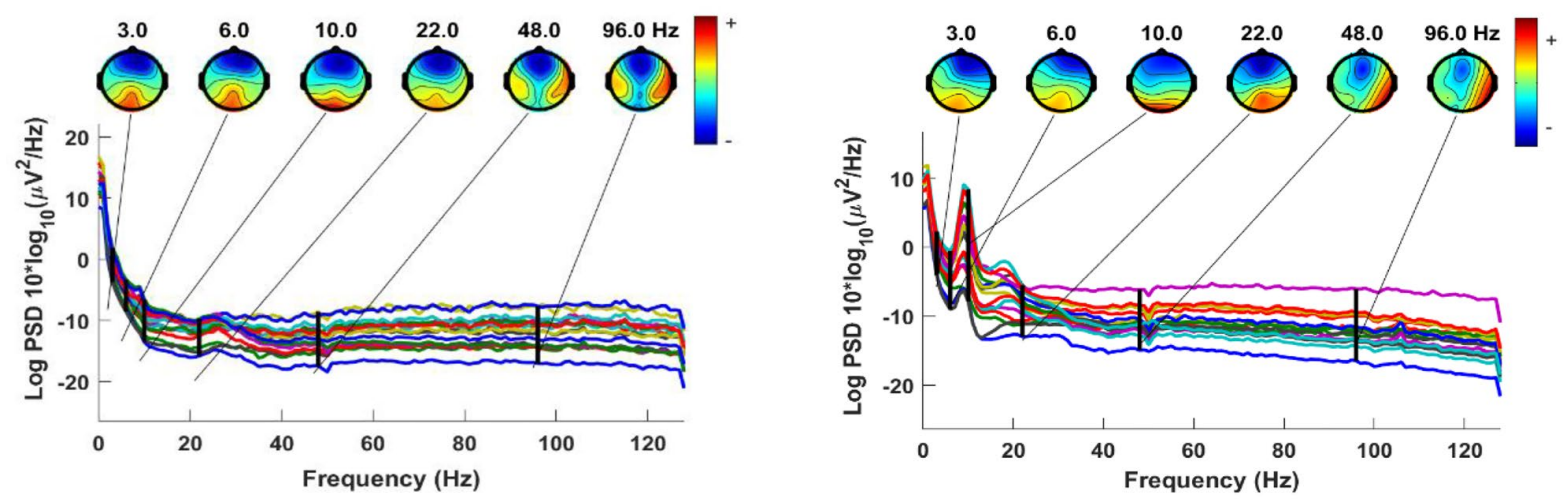
EEG PSD for a health control participant (left) and a participant with MCI (right).

The EEG data underwent pre-processing using the EEGLAB toolbox^43^. Initially, a band-pass filter was applied to the raw signals, with cut-off frequencies set at 0.5 Hz and 64 Hz. This filter effectively eliminated low-frequency drift and high-frequency noise. Subsequently, the data were re-referenced to the common average, further enhancing the quality of the signals. AC power line noise was then removed using Cleanline, an EEGLAB plugin. Independent component analysis (ICA) was used within EEGLAB to remove other artifacts, such as the electromyogram, electrocardiogram, and electrooculogram. Finally, the remaining artifacts were removed by visual inspection to ensure a clean dataset. Through visual inspection, we found the EEGs of three healthy subjects were still noisy, so they were excluded from our study, making the dataset almost balanced (11 MCI and 13 HC). Figure 3 illustrates an instance of PSD for the 19-channel EEGs of a healthy control and an individual with MCI. The figure also includes electrode maps for six selected frequencies: 3 Hz, 6 Hz, 10 Hz, 22 Hz, 48 Hz, and 96 Hz.

Next, the EEG signals are divided into equal segments, each containing multiple channels, with each segment having a size of $ch \times N$. Here, $ch$ represents the total number of channels, while $N$ corresponds to the number of samples per channel. We experimented with different values of segment lengths (2 s, 5 s, and 10 s) and found no significant difference in the performance of MCI detection. The noticeable difference was only the speed of execution of the signal processing and optimization processes. Therefore, we selected a 10-s segment length to reduce the processing time.

### Feature extraction (FE)

To extract features from a signal, we propose first to decompose it into sub-signals, each containing distinct subband frequencies. Subsequently, one feature is computed from each sub-signal (subband) by applying one measure. This approach enables the extraction of relevant information from different frequency components.

#### Signal decomposition

**Figure 4:**
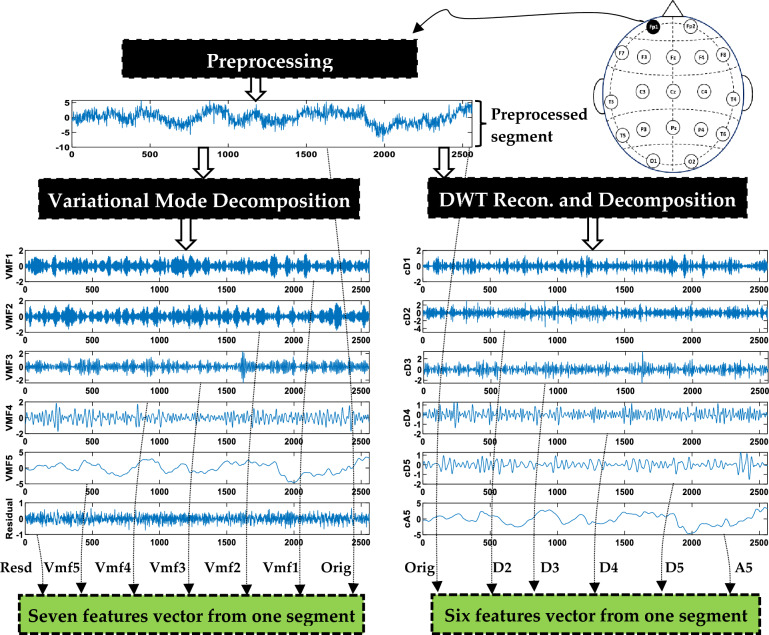
An illustrative instance of extracting features from a 10-s segment.

Typically, EEG signals are partitioned into five distinct subbands known as delta (less than 4 Hz), theta (4–8 Hz), alpha (8–13 Hz), beta (13–30 Hz), and gamma (greater than 30 Hz)^33^. Our previous studies^21,34,40^ have demonstrated that EEG signal decomposition contributes to producing effective features and significantly enhances classification accuracy. This is because the decomposition process can highlight information hidden in the data. Multiple decomposition methods are available for utilization. However, in this study, the focus is on selecting decomposition methods that offer a combination of simplicity and effectiveness. The objective is to develop feature extraction methods that are both efficient and highly effective. For this purpose, VMD and DWT are employed. The methodologies in^21,40^ are also adopted in the present study by decomposing each signal segment into either variational mode functions (VMFs) using VMD or approximation and detail coefficients using DWT. Detailed descriptions of these algorithms can be found in^21,39,44,45^. Figure 4 provides an illustrative instance of decomposing a segment by means of VMD and DWT. With VMD, the output comprises five VMFs and one residual signal. The central frequencies of the generated VMFs (VMF1 to VMF5) are 61.18 Hz, 41.27 Hz, 24.74 Hz, 11.01 Hz, and 1.25 Hz, respectively. These frequencies correspond to distinct EEG subbands. On the other hand, DWT generates an approximate coefficient (A5) and five detail coefficients (D1 to D5). These coefficients correspond to the subbands of 64–128 Hz, 32–64 Hz, 16–32 Hz, 8–16 Hz, 4–8 Hz, and 0–4 Hz, respectively.

#### Feature computation

The subsequent step, as illustrated in Fig. 4, involves the computation (extraction) of features from the obtained sub-signals. In this study, several measures are investigated for computing features: standard deviation (STD), interquartile range (IQR), band power (LBP), Teager energy (TeEng), Shannon entropy (ShEn), transformation-Shannon entropy (TShEn), sure entropy (SuEn), threshold entropy (ThEn), Katz's fractal dimension (KFD), and Higuchi's fractal dimension (HFD). These entropy, energy, and band power measures are defined in our previous works^21,40^, whereas Katz's and Higuchi's fractal dimensions are defined in^18^. It is worth noting that only one measure is used to compute the features. In other words, these measures are investigated individually.

When utilizing VMD, seven feature values are derived from each individual segment of a single channel, as illustrated in Fig. 4. These features include five from VMFs, one from the residual signal, and one from the original signal segment. If there are $ch$ channels, a feature vector of dimensions $7 \times ch$ is extracted for each multi-channel segment. With DWT, six feature values are extracted: one from A5, four from details (D2–D5), and the sixth from the original signal. D1 is excluded because applying a 0.5–64 Hz band pass filter in the preprocessing stage. The length of the resulting feature vector extracted for each segment is 6 × $ch$. The process in Fig. 4 is repeated over all multi-channel segments of all subjects’ data (MCI and HC) to obtain all feature vectors (feature matrix). The subsequent step involves implementing classification using the obtained feature vectors.

### Classification and performance assessment

In this study, the following techniques are investigated for the problem of classifying MCI versus HC: bagging-based RF, L/QDA, SVM, and KNN. A detailed examination of these classification techniques can be found in^46–49^. Classifiers’ parameters are optimized using NSGA-II to enhance the classification accuracy. RF is tested with various tree depths ranging from 1 to 35, while SVM is tested with three kernels: linear, polynomial, and radial basis function. For KNN, the number of neighbors is tested from 1 to 10. The hyperparameters of each classifier, along with the ones that are optimized, are summarized in Table 1.

**Table 1:** Hyperparameters of the classification methods.

Method	Hyperparameters
RF	Type of learner = ‘decision tree’, ensemble = ‘bag’, tree depths = (optimized: 1 to 35)
SVM	Type of kernel = (optimized: linear, polynomial, or radial basis function), method = ’least squares’, C = 2e-1
DA	Type of discriminant = (optimized: linear, or quadratic)
KNN	Number of neighbors = (optimized: 1 to 10), distance = ’optimized: ten types’, rule = ’nearest’

In order to assess the ability of each model to differentiate between persons with MCI and normal, we employ the LOSO CV. LOSO involves dividing the feature matrix, which consists of all the feature vectors, into multiple subsets. Each subset contains feature vectors belonging to a single subject. Since there are 24 subjects in our study, 24 subsets are created. Hence, 24 iterations are needed to complete the performance evaluation process. For each iteration, the feature vectors of a single subject are left out and used as the test set, while the feature vectors of the remaining subjects are used to train the model. This procedure is repeated 24 times to ensure that the data from each subject is utilized as a test set precisely once. This approach provides a realistic and reliable estimate of a model's performance by systematically evaluating it across different subjects and ensuring that no subject's data is used simultaneously in the training and test sets^50^. At each iteration, the classification accuracy (CA) is calculated from a test set using the following equation:

1 $$CA = \frac{{N_{{{\text{correct}}}} }}{{N_{{{\text{total}}}} }} \times 100\%$$

where $N_{{{\text{total}}}}$ is the total number of feature vectors in the test set, and $N_{{{\text{correct}}}}$ represents the number of feature vectors that are correctly classified. To evaluate the model’s performance, a single classification accuracy score is obtained by averaging the obtained accuracy scores over the 24 iterations. Similarly, sensitivity, specificity, precision, and F-score can be computed using Eqs. (2) to (5) at each iteration, and the results are then averaged over the 24 iterations.

2 $$Sensitivity = \frac{TP}{{TP + FN}} \times 100\%$$

3 $$Specificity{ } = \frac{TN}{{TN + FP}} \times 100\%$$

4 $$F - score{ } = 2 \times \frac{{Precision{ } \times Sensitivity}}{Precision + Sensitivity}$$

where $TP$ stands for true positives, $TN$ for true negatives, $FN$ for false negatives, and $FP$ for false positives. Sensitivity measures the ability of a classifier to accurately identify individuals with the condition, while specificity measures the ability of the classifier to correctly identify individuals without the condition^51^. The number of accurate positive predictions made is measured by the *precision* metric, which is defined as

5 $$Precision{ } = \frac{TP}{{TP + FP}} \times 100$$

### Channel and feature selection

EEG channel and feature selection play a crucial role in mitigating the computational burden of signal processing and enhancing classification accuracy by eliminating redundant or irrelevant information. In this study, the NSGA is applied to selecting channels and features with the aim of enhancing classification performance. In the following, NSGA is briefly discussed, along with an overview of the problems to be tackled.

#### NSGA algorithm

The genetic algorithm (GA) takes inspiration from Charles Darwin's natural evolution theory as a basis for its functioning. A population in GA is made up of a group of potential solutions known as chromosomes, each of which represents a collection of parameters known as genes. GA incorporates a range of tactics aimed at generating optimal solutions, encompassing population initialization, computation of the fitness function, crossover, mutation, survivor selection, and criteria for termination^51^.

In optimization problems, particularly ones with many objectives, the pareto-optimal solution—also called a non-dominated solution—performs better than all others. NSGA's initial version uses a niche strategy to preserve stable sub-populations of high-quality solutions known as the Pareto front. By employing a non-dominated sorting and selection procedure, this method emphasizes potential candidates^52^. NSGA-II, the NSGA's second version, was released to solve various shortcomings of the initial version, including population variety, non-elitist methods, and computing complications^53^. The specifics of NSGA methods are outside the scope of this study, and readers are referred to^52,53^. In a previous study^40^, we explored different EEG channel selection methods and concluded that NSGA-II offers a considerable performance improvement over other techniques. Therefore, we adopt the NSGA-II method for channel selection in this study.

#### Optimization problems and variable definition

NSGA-II is employed to tackle two problems, each of which has two objective functions. The first problem is to enhance classification accuracy while simultaneously lowering the quantity of EEG channels. On the other hand, the second problem centers around lowering the quantity of features while simultaneously enhancing classification accuracy. The problems are presented in generic form in Eqs. (6) and (7):

6 $${\text{The}}\;{\text{first}}\;{\text{problem}}:\left\{ {\begin{array}{*{20}l} {{\text{Minimize}}} \hfill & {No\_ch} \hfill \\ {{\text{Maximize}}} \hfill & { averag\; CA \left( {channels, Params} \right)} \hfill \\ {{\text{Subject}}\;{\text{to}}} \hfill & { No\_ch \ge 1} \hfill \\ {} \hfill & { averag\; CA \le 100} \hfill \\ \end{array} } \right.$$

7 $${\text{The}}\;{\text{ second}}\;{\text{ problem}}:\left\{ {\begin{array}{*{20}l} {{\text{Minimize}}} \hfill & {No\_feat} \hfill \\ {{\text{Maximize}}} \hfill & {averag\; CA \left( {features, Params} \right)} \hfill \\ {{\text{Subject}}\;{\text{ to}}} \hfill & {No\_feat \ge 1} \hfill \\ {} \hfill & {averag\; CA \le 100(\% )} \hfill \\ \end{array} } \right.$$

where $No\_ch$ is the number of EEG channels, while $No\_feat$ is the number of features. The average classification accuracy is denoted by $averag CA$, and $Params$ represents parameters for the classifier.

**Figure 5:**
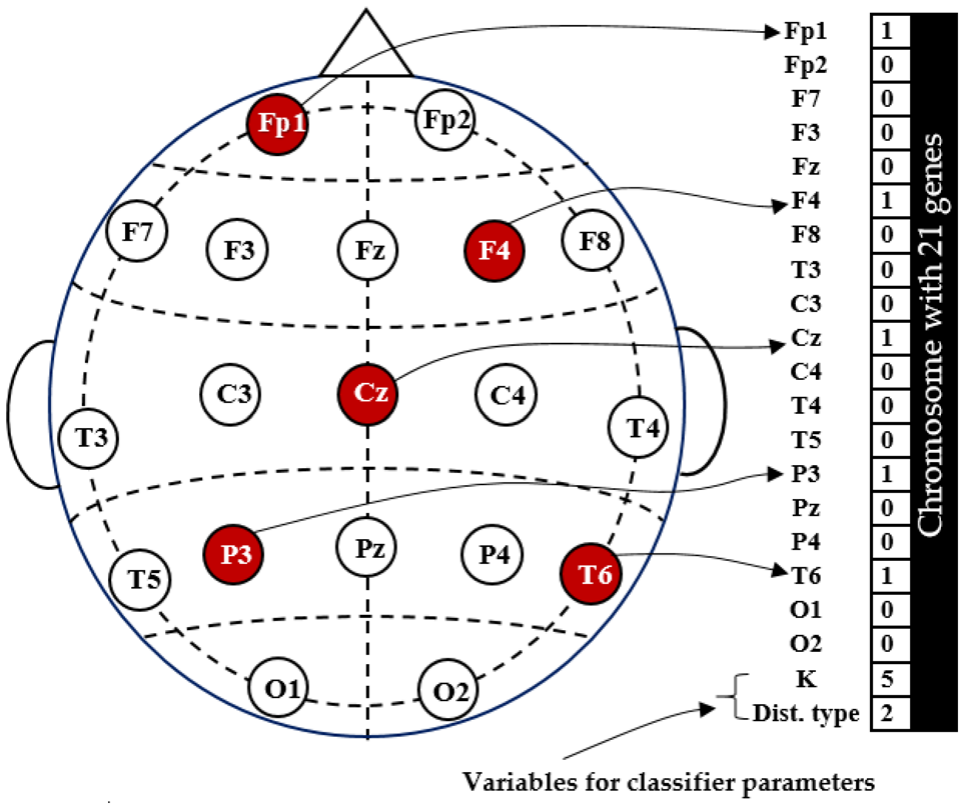
An illustration of how channels and classifier parameters are represented within a chromosome.

The implementation of NSGA-II requires a proper representation of all variables involved in the above optimization problems. For the first problem (channel selection), as shown in Fig. 5, a chromosome with 21 genes (21 variables) is required to represent all channels $ch_{1}$, $ch_{2}$,…,$ch_{19}$, in addition to two classifier parameters. The channel variables are binary, having two possible values: 1 or 0. A value of 1 signifies that the channel is chosen for the classification, whereas a value of 0 signifies that the channel is not chosen. The final two variables store the optimized parameter values of the classifier. A similar variable representation is applied for the second problem (feature selection). In the case of DWT, a 6 × ch feature vector is represented by 114 genes (an example is shown in Fig. 5), while in the case of VMD, a 7 × ch feature vector is represented by 133 genes. One or two variables are appended to the chromosome to represent the classifier’s parameters that need to be optimized.

**Figure 6:**
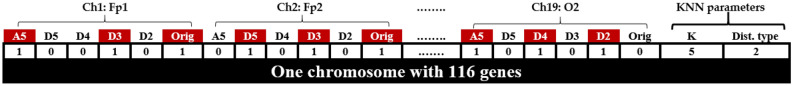
Illustrative instance of how DWT features and classifier parameters are represented within a chromosome.

The number of variables required to represent parameters is different for each classifier. In the case of KNN, two variables are required, as shown in Figs. 5 and 6. The first variable is for the number of neighbors (K) and can take values of 1, 2,…, or 10, while the second is for distance type: 1 for ‘euclidean’, 2 for ‘seuclidean’, 3 for ‘cityblock’, 4 for ’chebychev’, 5 for ’minkowski’, 6 for ’mahalanobis’, 7 for ’cosine’, 8 for ’correlation’, 9 for ’spearman’, and 10 for ‘hamming’. Regarding the SVM classifier, a single variable is employed to designate the kernel type, with the options being: 1 for linear, 2 for polynomial, and 3 for RBF. Similarly, when representing the type of DA, the value 1 corresponds to linear, and 2 corresponds to quadratic. Regarding the RF, $Param$ denotes the tree depth, with permissible values spanning from 1 to 35.

**Figure 7:**
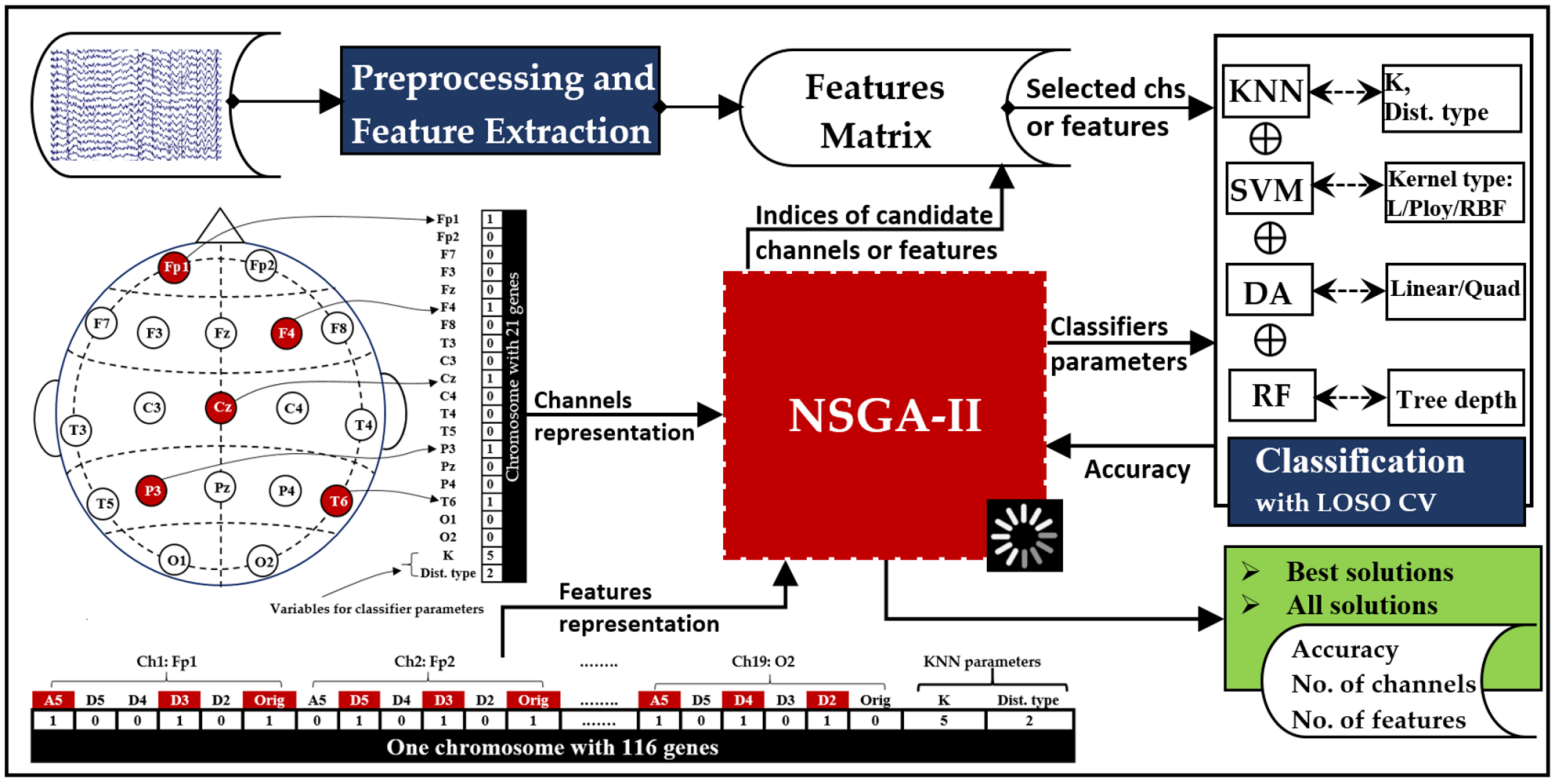
The complete process of MCI detection with the use of NSGA for the EEG channel, feature selection, and the classifier's parameter optimization.

Figure 7 illustrates the complete process, which consists of several phases. The process can be summarized in the following few lines. After reading the raw signals, they undergo the preprocessing stage and are then subjected to either DWT or VMD decomposition, as previously illustrated (see Fig. 4). Next, features are computed using one of the adopted measures, namely STD, IQR, LBP, TeEng, ShEn, TShEn, SuEn, ThEn, KFD, or HFD. The computed features are then organized into a feature matrix and stored for subsequent iterative process. The next phase is applying NSGA-II to extensively explore a minimum subset of channels (or features) and fine-tune the classifiers' parameters to maximize classification accuracy. The NSGA-II algorithm commences by generating an initial population comprising a diverse range of solutions (chromosomes). When it comes to channel selection, the classification procedure exclusively considers the features associated with channels signified by "1", while rigorously excluding the features belonging to channels signified by “0”. In other words, when a channel is represented by 1, all features belonging to that channel are included in the classification. On the other hand, when it comes to feature selection, each feature within a channel is represented by one value (0 or 1), and only the features that are represented by 1 are considered. In the classification stage, the average $CA$ is computed using LOSO CV. During this phase, NSGA-II employs $CA$ and $No\_ch$ (or $No\_feat$), to assess each solution present within the current population. NSGA-II proceeds by generating a new population and evaluating the solutions, leading to the gradual evolution of a population comprised of potential solutions. This iterative process persists until a maximum number of iterations $\left( {MaxIter} \right)$ is reached. In the channel selection problem, the population size is set at 200 and $MaxIter$ is 50. The population size of the feature selection problem is configured as 1000 and $MaxIter$ is 100. The implementation of all operations depicted in Fig. 7 is carried out using Matlab 2022.

## Results

As described earlier, all EEG signals are first preprocessed and then split into 10-s multi-channel segments. Each participant contributes 60 segments, resulting in a total of 1440 segments from all participants. With VMD, each segment is converted into a feature vector consisting of 133 elements, whereas DWT yields a feature vector of length 114, gathered from 19 channels. Using the LOSO method, the resulting feature vectors are used for classifier training and testing. The effectiveness of the adopted channel and feature selection method is demonstrated using three experiments:

Experiment I: classification using all channels and features,

Experiment II: classification after selecting a subset of EEG channels, and

Experiment III: classification after selecting a subset of features.

### Full-channels-based results (experiment I)

In this experiment, we evaluate the MCI vs. HC classifier performance when all EEG channels are considered. That means all features in each feature vector are included in the classification. Table 2 shows the average LOSO classification accuracy of four classifiers using VMD or DWT combined with one of the ten non-linear measures mentioned in “Feature extraction (FE)” Section. In this experiment, it is observed that the DWT-based results generally outperform the VMD-based results in most cases. The highest accuracy achieved is 89.72% using the DWT-STD-LDA combination. For comparison, similar results but with tenfold cross-validation are presented in Table 3. The results in Table 2 are considerably worse. For example, the VMD-KFD-KNN combination achieved an accuracy of 53.26% with LOSO, but with tenfold, the estimated accuracy is 94.03%. This is anticipated as the LOSO ensures that data from one participant isn't used simultaneously in the test and training groups. In contrast, the tenfold (or, in general, k-fold) approach divides the data so that the data of the same participant is included in both training and testing groups, causing the accuracy scores to be overestimated. Because LOSO avoids this form of data leakage, it is adopted in this study. However, it is worth noting that most of the LOSO accuracy scores reported in Table 2 are not deemed satisfactory, especially those obtained using RF and KNN classifiers. Therefore, in the following experiments, we aim to improve the classification accuracy by selecting subsets of EEG channels and features.

**Table 2:** LOSO classification accuracy with the use of all channels.

Measures	VMD				DWT			
	RF_NT=30_	LDA	SVM_Linear_	KNN_K=3_	RF_NT=30_	LDA	SVM_Linear_	KNN_K=3_
STD	63.19	85.21	80.83	68.06	64.86	89.72	88.96	65.56
IQR	63.47	82.22	80.07	67.15	67.36	88.26	81.67	66.81
LBP	64.51	79.51	76.88	63.61	64.68	79.79	86.39	63.40
TeEng	72.99	79.58	74.24	55.28	68.33	80.35	80.14	58.54
ThEn	65.35	70.42	73.19	55.28	68.54	75.42	70.62	53.75
SuEn	64.03	77.85	75.07	62.36	63.75	85.63	85.00	63.47
ShEn	61.25	70.49	72.85	69.24	66.60	85.62	77.15	68.47
TShEn	62.22	70.14	68.82	61.11	65.07	76.81	74.39	60.76
HFD	54.79	54.58	54.17	49.93	56.74	62.99	61.94	51.46
KFD	74.24	74.65	77.36	53.26	72.15	86.46	84.31	54.51

**Table 3:** Tenfold classification accuracy with the use of all channels.

Measures	VMD				DWT			
	RF_NT=30_	LDA	SVM_Linear_	KNN_K=3_	RF_NT=30_	LDA	SVM_Linear_	KNN_K=3_
STD	95.21	94.65	93.96	94.44	95.51	95.42	95.49	94.24
IQR	94.72	95.00	94.03	93.68	93.54	95.07	95.42	92.99
LBP	95.14	95.28	94.79	94.65	94.93	95.76	95.76	93.40
TeEng	94.51	95.56	95.42	94.44	94.79	95.56	95.76	93.75
ThEn	95.00	95.35	94.72	93.54	94.86	95.69	95.63	93.13
SuEn	95.00	95.28	94.58	94.93	94.03	95.14	95.69	93.33
ShEn	94.65	92.78	93.61	93.61	94.17	93.96	94.24	92.85
TShEn	95.35	95.14	94.03	94.58	94.72	95.56	95.28	93.54
HFD	91.94	90.07	89.58	86.39	92.99	92.08	91.67	93.13
KFD	94.10	94.65	94.24	94.03	94.72	95.07	95.35	93.06

### EEG channel selection-based results (experiment II)

In this experiment, we adopt the NSGA-II algorithm to select a subset of EEG channels. In this case, the classification includes the features that belong to the channels that were chosen but leaves out the features that belong to the channels that weren't chosen. The classifier parameters are also optimized using NSGA-II. In this subsection, KNN-based results are presented first, and the results based on other classifiers are discussed later. Tables 4 and 5 show the KNN classification accuracy using VMD and DWT, respectively, each combined with six selected measures. The tables show that the classification accuracy scores are improved when selecting a few suitable channels. For example, in Table 4, the VMD-KFD-KNN classification accuracy when using all channels is 53.26%. When NSGA-II is applied to select two or three channels, the accuracy improves to 77.36% and 87.22%, respectively. Another example from Table 5: the DWT-ThEn-KNN combination with NSGA-II achieved accuracy scores of 83.75% and 87.86% with two and three selected channels, respectively, while the accuracy of using all channels is 53.75%. The dash ‘-’ in Tables 4 and 5 indicates that no subset of channels is returned by NSGA-II as it couldn’t find the optimal subset of channels that achieved higher accuracy than that achieved by the preceding smaller subset. Regarding the classifier parameters, NSGA-II optimizes the results by selecting the two parameters of the KNN classifier: number of neighbors (K) and distance type. In this experiment, the most selected value of K is 5, followed by 3. On the other hand, there was no particular distance type that was consistently chosen by NSGA-II in most solutions.

**Table 4:** The classification accuracy of the KNN with channel selection (VMD-based FE methods).

KNN	Classification accuracy (selected value of k, distance type)					
No. of channels	VMD + IQR	VMD + LBP	VMD + TeEng	VMD + SuEn	VMD + ShEn	VMD + KFD
1	68.54 (5,7)	65.63 (5,8)	58.26 (5,2)	61.11 (5,3)	61.81 (3,3)	57.43 (5,6)
2	78.89 (5,3)	79.17 (5,3)	81.32 (5,2)	78.06 (3,2)	79.31 (5,4)	77.36 (5,7)
3	82.99 (5,1)	84.24 (3,2)	**86.32** (5,2)	82.99 (5,7)	85.56 (5,1)	**87.22** (5,8)
4	**85.07** (5,4)	**85.90** (3,2)	–	**86.04** (3,7)	85.69 (5,3)	–
5	–	–	–	–	**85.90** (3,1)	–
All channels	67.15 (3,1)	63.61 (3,1)	55.28 (3,1)	62.36 (3,1)	69.24 (3,1)	53.26 (3,1)

Significant values are in [bold].

**Table 5:** The classification accuracy of the KNN with channel selection (DWT-based FE methods).

KNN	Classification accuracy (selected value of k, distance type)					
No. of channels	DWT + STD	DWT + TeEng	DWT + ThEn	DWT + SuEn	DWT + TShEn	DWT + KFD
1	68.96 (5,7)	62.50 (3,8)	57.64 (1,2)	60.21 (5,6)	61.18 (3,7)	59.17 (5,6)
2	81.88 (5,3)	**85.35** (3,2)	83.75 (5,2)	79.58 (5,2)	79.10 (5,2)	80.21(5,7)
3	84.24 (5,1)	–	**87.36** (5,2)	85.00 (5,2)	82.85 (3,2)	–
4	**86.81** (5,1)	–	–	**86.18** (5,1)	83.19 (3,2)	83.47 (5,2)
5	–	–	–	–	–	–
6	–	–	–	–	–	**83.89** (1,8)
All channels	65.56 (3,1)	58.54 (3,1)	53.75 (3,1)	63.47 (3,1)	60.76 (3,1)	54.51 (3,1)

Significant values are in [bold].

Although there is a significant improvement when a few appropriate channels are selected, the accuracy scores still need to be improved, as the highest accuracy obtained is 87.22%. Therefore, in the third experiment, we investigate the use of NSGA-II for feature selection.

### Feature selection-based results (experiment III)

This experiment is conducted independently from Experiment II. In other words, the feature selection process considers all of the 19 EEG channels and not the subset of channels obtained in Experiment II. As discussed in “Feature extraction (FE)” Section, each segment within a channel is decomposed into different subbands, and one feature is extracted from each subband. In this experiment, it is not a requirement for all features (subbands) within a specific channel to be selected. It is possible that only some of the features or even none of them are chosen for the classification. In addition, it is possible that features or subbands that are selected for a particular channel are different from those of another channel. Table 6 shows the classification results when VMD and various measures are used. The first column in the table presents the number of features selected, while the other columns include the corresponding accuracy for each measure. For comparison purposes, the last row in the table contains the classification accuracy when all features collected from 19 channels are included in the classification. There is a significant improvement over all measures due to using NSGA-II for feature selection. For instance, with the VMD-KFD-KNN combination, the accuracy obtained with all 133 features collected from all channels is 53.26%, while with 19 features selected from 11 channels, the accuracy is improved to 92.57%. Another example is with VMD-SuEn-KNN, where the accuracy when only ten of the 133 features were selected is 91.53%, which is significantly better than the 62.36% accuracy obtained when all features are used.

**Table 6:** The classification accuracy of the KNN with feature selection (VMD-based FE methods).

No. of Features	Classification accuracy % (no. of channels)					
	VMD + KFD	VMD + TeEng	VMD + SuEn	VMD + LBP	VMD + IQR	VMD + ShEn
1	–	61.94	65.42	55.21	57.22	–
2	73.75	87.01	81.39	80.35	80.97	–
3	81.67	88.68	84.10	84.79	84.72	82.92
4	87.57	91.32	88.26	86.39	87.78	84.58
5	89.31	91.94	–	88.33	88.13	85.55
6	90.83	92.22	88.89	88.96	89.24	85.83
7	91.73	–	90.21	90.42	–	86.67
8	91.74	92.29	90.97	90.90	–	88.06
9	92.22	92.43	91.11	91.11	–	88.40
10	–	–	**91.53 (6)**	91.25	89.51	88.47
11	–	92.57	–	91.39	–	88.68
12	–	**92.71 (6)**	–	91.53	89.65	88.96
13	–	–	–	91.67	**89.86 (9)**	-
14	–	–	–	–	–	**89.10 (8)**
15	–	–	–	91.74	–	–
16	92.29	–	–	–	–	–
17	92.36	–	–	–	–	–
18	–	–	–	91.88	–	–
19	**92.57 (11)**	–	–	–	–	–
20	–	–	–	**91.94 (11)**	–	–
All (133)	53.26 (19)	55.28 (19)	62.36 (19)	63.61 (19)	67.15 (19)	69.24 (19)

Significant values are in [bold].

For more investigation, the current experiment is repeated but using DWT, and the results are presented in Table 7. Similar to the VMD results, the DWT accuracy results in Table 7 demonstrate significant improvement when a few suitable features are selected. For example, in the case of the DWT-STD-KNN combination, the accuracy obtained with all 114 features collected from all 19 channels is 65.56%, while with 13 features selected from 8 channels, the accuracy increases to 91.04%.

**Table 7:** The classification accuracy of the KNN with feature selection (DWT-based FE methods).

No. of Features	Classification accuracy % (no. of channels)					
	DWT + STD	DWT + ThEn	DWT + SuEn	DWT + TShEn	DWT + KFD	DWT + TeEng
1	55.63	–	65.28	64.65	60.07	60.21
2	80.83	60.49	81.60	81.67	75.07	86.04
3	86.32	87.85	87.15	86.39	81.67	88.68
4	89.86	88.26	89.93	89.03	86.04	91.11
5	90.28	89.79	91.32	90.07	89.86	91.67
6	90.42	90.49	91.46	91.11	–	92.08
7	90.76	90.69	91.74	–	90.63	92.15
8	–	–	91.88	–	91.32	92.36
9	–	**91.39 (6)**	92.22	–	91.67	92.64
10	–	–	–	–	–	**93.13 (6)**
11	90.90	–	–	91.67	–	–
12	90.97	–	92.29	-	–	–
13	**91.04 (8)**	–	–	91.74	92.01	–
14	–	–	92.71	–	–	–
15	–	–	–	–	92.15	–
16	–	–	**92.92 (9)**	–	92.29	–
17	–	–	–	**91.81 (9)**	**92.50 (10)**	–
All (114)	65.56 (19)	53.75 (19)	63.47 (19)	60.76 (19)	54.51 (19)	58.54 (19)

Significant values are in [bold].

### Results using other classifiers

The previous results of experiments II and III were obtained using only the KNN classifier. In this subsection, we use different classifiers to demonstrate the efficiency of the suggested EEG channel and feature selection strategy. Tables 8 and 9 present the outcomes of EEG channel selection using RF, SVM, DA, and KNN classifiers. Table 8 includes the results of 24 combinations based on VMD, while Table 9 shows similar results using DWT. The numbers enclosed in square and round brackets indicate the selected channels and the parameters, respectively. When comparing the results obtained using all channels (see Table 2) with the results presented in Tables 8 and 9, a clear improvement in classification accuracy scores can be observed across all measures and classifiers. For example, in the VMD-KFD-SVM combination, the classification accuracy obtained when all channels are used is 77.36%, while an accuracy of 90.49% is obtained when seven channels are selected by NSGA-II. Similarly, when using only six channels, the DWT-TeEng-LDA combination achieves an accuracy score of 92.78%, while the accuracy with all channels is 80.35%. Among the outcomes in Tables 8 and 9, the highest accuracy of 94.86% is obtained by the DWT-ThEn-LDA combination when seven channels are selected by NSGA-II.

**Table 8:** The optimal classification accuracy due to channel selection (VMD-based FE methods).

FE method	Accuracy (selected parameter) [selected channels]			
	RF	SVM	DA	KNN
VMD + KFD	85.49 (22) [Fp1, F8, Cz, T4, Pz]	90.49 (linear) [Fp1, F8, T3, Cz, C4, T4, P3]	88.68 (linear) [Fp1, C3, Cz, T4, P3, T6]	87.22 (5,8) [Fp1, Cz, Pz]
VMD + TeEng	86.32 (29) [F8, C3, Cz, T4]	91.56 (linear) [Fp1, F8, Cz, T4, Pz]	91.94 (linear) [F8, C3, Cz, T4, T6]	86.32 (5,2) [Fp1, F8, Cz]
VMD + SuEn	81.74 (33) [Fp1, F8, Cz]	89.72 (linear) [Fp2, F8, Cz, T4, P4]	89.58 (linear) [F7, F8, C3, Cz, T4, T5, T6]	86.04 (3,7) [Fp1, F8, Cz, T4]
VMD + LBP	80.56 (33) [Fp1, F8, Cz]	90.00 (linear) [Fp2, F8, Cz]	90.97 (linear) [C3, Cz, P3, Pz, T6]	85.69 (3,2) [Fp1, F8, Cz]
VMD + IQR	82.64 (31) [Fp2, F8, Cz]	90.76 (linear) [Fp1, F7, C3, Cz, T4, T5, P4, T6]	90.21 (linear) [F7, F4, F8, C3, Cz, C4, T4, T6]	85.07 (5,4) [Fp1, F8, Cz, T4]
VMD + ShEn	80.42 (29) [Fp2, Fz, F8, Cz]	85.28 (linear) [Fp1, Cz, T4, Pz, P4, O2]	80.97 (linear) [Fp1, F8, C3, Cz, T4, P3, T6, O2]	86.67 (5,1) [Fp1, F8, C3, Cz, C4]

**Table 9:** The optimal classification accuracy due to channel selection (DWT-based FE methods).

FE method	Accuracy (selected parameter(s)) [selected channels]			
	RF	SVM	DA	KNN
DWT + STD	88.05 (31) [F8, Cz, T4, Pz]	93.54 (1) [Fp1, Fp2, F7, Fz, Cz, T4, P4, T6]	92.64 (1) [Fp1, Fp2, C3, Cz, T4, T5, P3]	86.81 (5,1) [Fp1, F8, Cz, T4]
DWT + ThEn	87.64 (35) [F8, C3, Cz]	94.86 (1) [F3, F8, Cz, C4, T4, T5, T6]	94.65 (1) [Fz, F4, F8, Cz, C4, T4, T5, T6]	87.36 (5,2) [Fp2, F8, Cz]
DWT + SuEn	87.43 (31) [F8, C3, Cz]	92.85 (1) [Fp1, Fp2, F7, F8, Cz, T4, T6]	92.15 (1) [F8, C3, Cz, T4, T5, P3, T6, O1]	86.18 (5,1) [Fp1, F8, Cz, T4]
DWT + TShEn	86.18 (28) [F8, Cz]	91.81 (1) [Fp1, Cz, T4, T5, P4]	91.94 (1) [F8, C3, Cz, T4, T5, T6, O1]	83.19 (3,2) [Fp1, F8, Cz, P4]
DWT + KFD	88.61 (37) [F8, Cz, T4]	91.32 (1) [Fp1, Cz, T4, P3, P4]	90.42 (1) [Fp1, F3, Cz, T4, T5, P3, P4, T6]	83.19 (1,8) [Fp1, F3, F8, Cz, C4, T4]
DWT + TeEng	87.99 (34) [F8, Cz, C4, T4]	92.64 (1) [Fp1, F8, Cz, T4, T6]	92.78 (1) [F8, C3, Cz, T4, T6, O2]	85.35 (3,2) [F8, Cz]

In the feature extraction case (Experiment III), Table 10 (VMD-based methods) and Table 11 (DWT-based methods) show the optimal classification accuracy scores for the 48 combinations of methods. The tables also present how many features are chosen for each combination and how many channels those features belong to. The feature selection results in Tables 10 and 11 show an additional improvement in accuracy when compared to the channel selection results (Tables 8 and 9). For instance, in the VMD-KFD-SVM combination, an accuracy of 77.36% is achieved when all channels are considered. A higher accuracy, 90.49%, is obtained once seven suitable channels are chosen with all of their features. A further improvement is observed when only 13 features belonging to 11 channels are chosen, leading to an accuracy of 93.89%. Another example from Table 11: the DWT-TeEng-LDA combination achieves an accuracy of 80.35% when all channels are included for the classification, an accuracy of 92.78% when six channels are selected, and an accuracy of 95.83% when 12 features belonging to 9 channels are selected. Within the results presented in Tables 10 and 11, four combinations based on DWT achieve the highest classification accuracy of 95.83% (highlighted in bold in Table 11).

**Table 10:** The optimal classification accuracy due to feature selection (VMD-based FE methods).

FE method	Accuracy (no. of selected features, no. of channel)			
	RF	SVM	DA	KNN
VMD + KFD	90.83 (14, 8)	93.89 (13, 11)	92.99 (17, 10)	92.57 (19, 11)
VMD + TeEng	88.82 (14, 9)	**95.28** **(8, 7)**	**95.63** **(15, 10)**	92.71 (12, 6)
VMD + SuEn	87.29 (10, 7)	94.51 (13, 9)	94.79 (19, 14)	91.53 (10, 6)
VMD + LBP	87.50 (6, 5)	95.00 (21, 13)	**95.48** **(19, 13)**	91.94 (20, 11)
VMD + IQR	88.54 (6, 5)	94.38 (17, 11)	94.31 (17, 11)	89.86 (13, 9)
VMD + ShEn	88.19 (10, 9)	92.50 (23, 13)	90.14 (23, 14)	89.10 (14, 8)

**Table 11:** The optimal classification accuracy due to feature selection (DWT-based FE methods).

FE method	Accuracy (no. of selected features, no. of channel)			
	RF	SVM	DA	KNN
DWT + STD	91.18 (9, 5)	95.56 (14, 11)	95.42 (8, 8)	91.04 (13, 8)
DWT + ThEn	90.63 (9, 6)	**95.83 (12, 8)**	95.63 (13, 8)	91.39 (9, 6)
DWT + SuEn	90.28 (14, 10)	**95.83 (10, 8)**	95.76 (9, 8)	92.92 (16, 9)
DWT + TShEn	90.35 (12, 6)	95.35 (13, 9)	95.42 (14, 10)	91.81 (17, 9)
DWT + KFD	90.14 (18, 12)	95.69 (11, 10)	95.00 (12, 10)	92.50 (17, 10)
DWT + TeEng	90.14 (12, 8)	**95.83 (9, 8)**	**95.83 (12, 9)**	93.13 (10, 6)

## Discussion

The present study focuses on selecting EEG channels (and features) using a multi-objective optimization method and computing the accuracy results based on the LOSO CV. The goal is to progress toward building an accurate MCI detection system with a small number of electrodes and features. For the purpose of demonstrating the usefulness of using the multi-objective NSGA-II optimization method for selecting EEG channels and features, three experiments have been conducted. In the first experiment, all channels and features were used for classification. The second experiment uses NSGA-II to select a subset of EEG channels. The third experiment uses NSGA-II to select suitable features belonging to a number of channels. To make the investigation general, two decomposition methods (VMD and DWT) that each produce signals with different frequency bands, various measures, and four classifiers are employed, building a lot of combinations (48 models).

**Figure 8:**
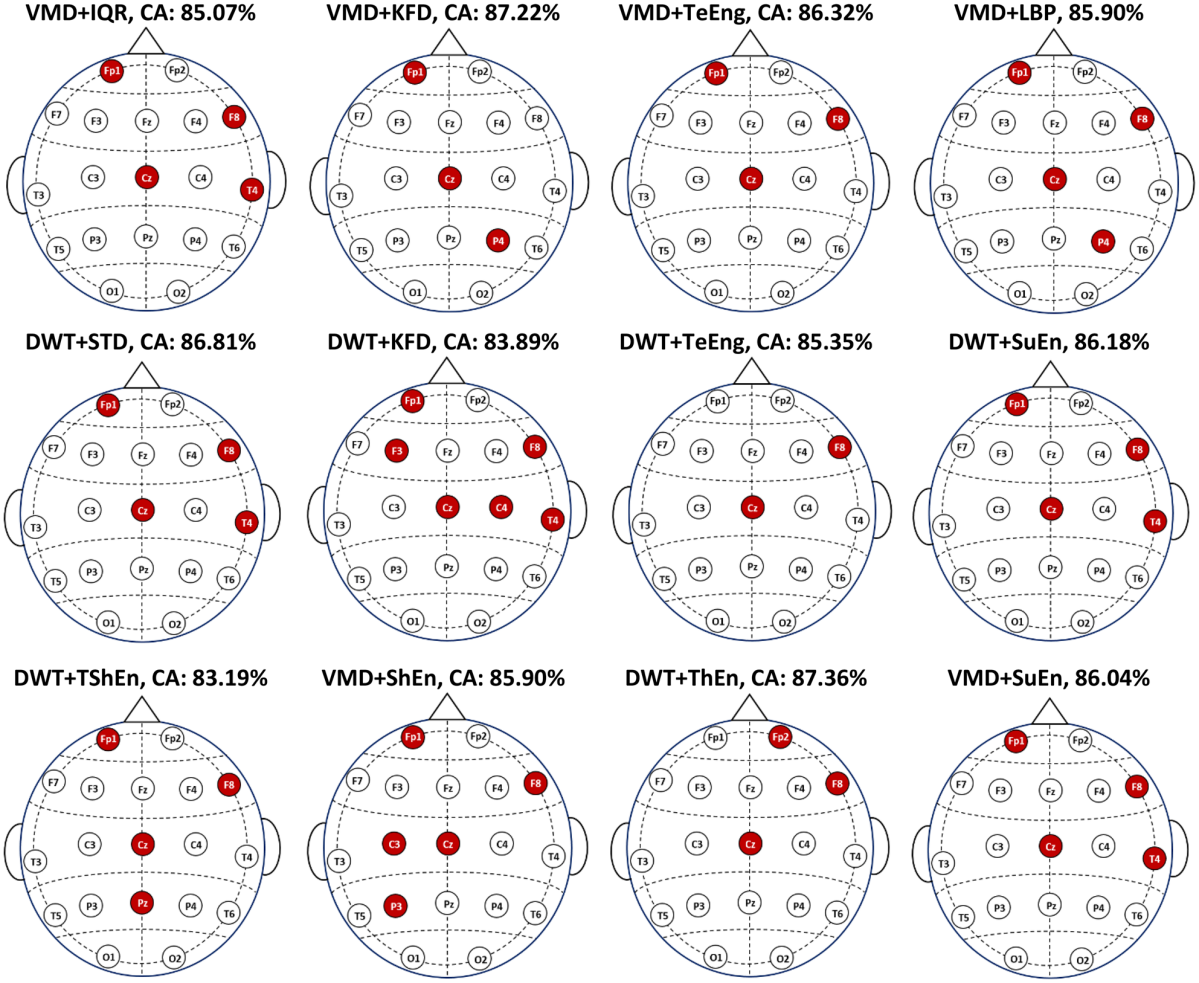
The selected channels that produce the maximum classification accuracy with KNN and twelve FE methods.

By comparing the results of Experiment I (Table 2) with those of Experiment II (Tables 4 and 5), the classification accuracy scores are improved when selecting a few suitable channels. This pattern of improved accuracy with channel selection can be observed across other combinations listed in Tables 4 and 5. These outcomes are in line with the studies^54,55^ that reviewed EEG channel selection for different tasks and concluded that selecting informative channels while excluding noisy or irrelevant ones reduces the impact of noise and results in improved accuracy. To illustrate the specific channels involved, Fig. 8 displays the channel topographies corresponding to the best solution (an optimal subset of channels) for each feature extraction method that yields the maximum classification accuracy. The figure shows that VMD + IQR, DWT + STD, DWT + SuEn, and VMD + SuEn feature extraction methods lead to identical solutions with channels Fp1, F8, Cz, and T4. Other feature extraction methods lead to different solutions, as each method may extract unique biomarkers. However, as shown in the figure, some channels, such as Fp1, F8, and Cz, appear in most solutions.

**Figure 9:**
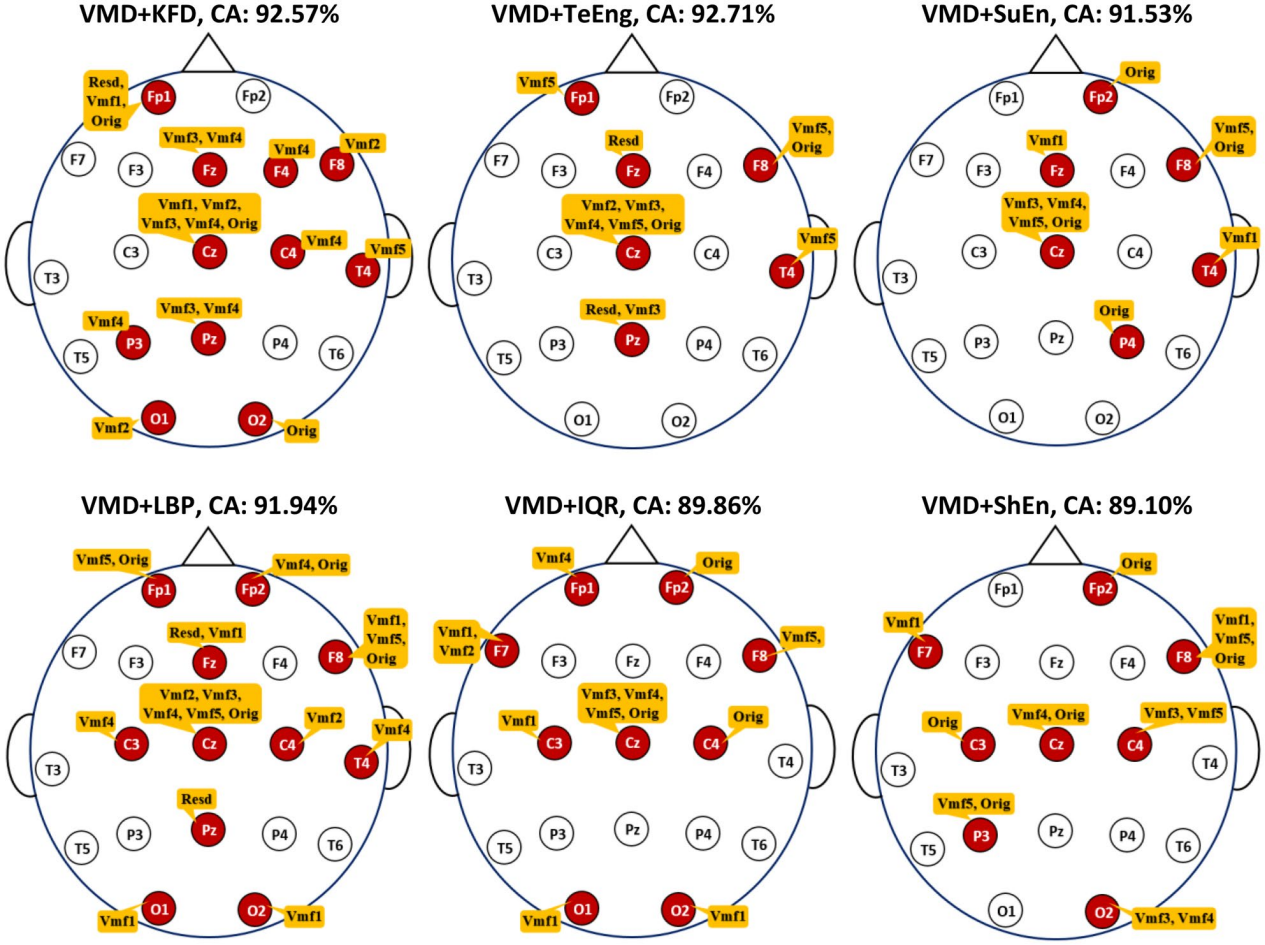
The optimal subsets of features and the channels they belong to when KNN and VMD are used.

**Figure 10:**
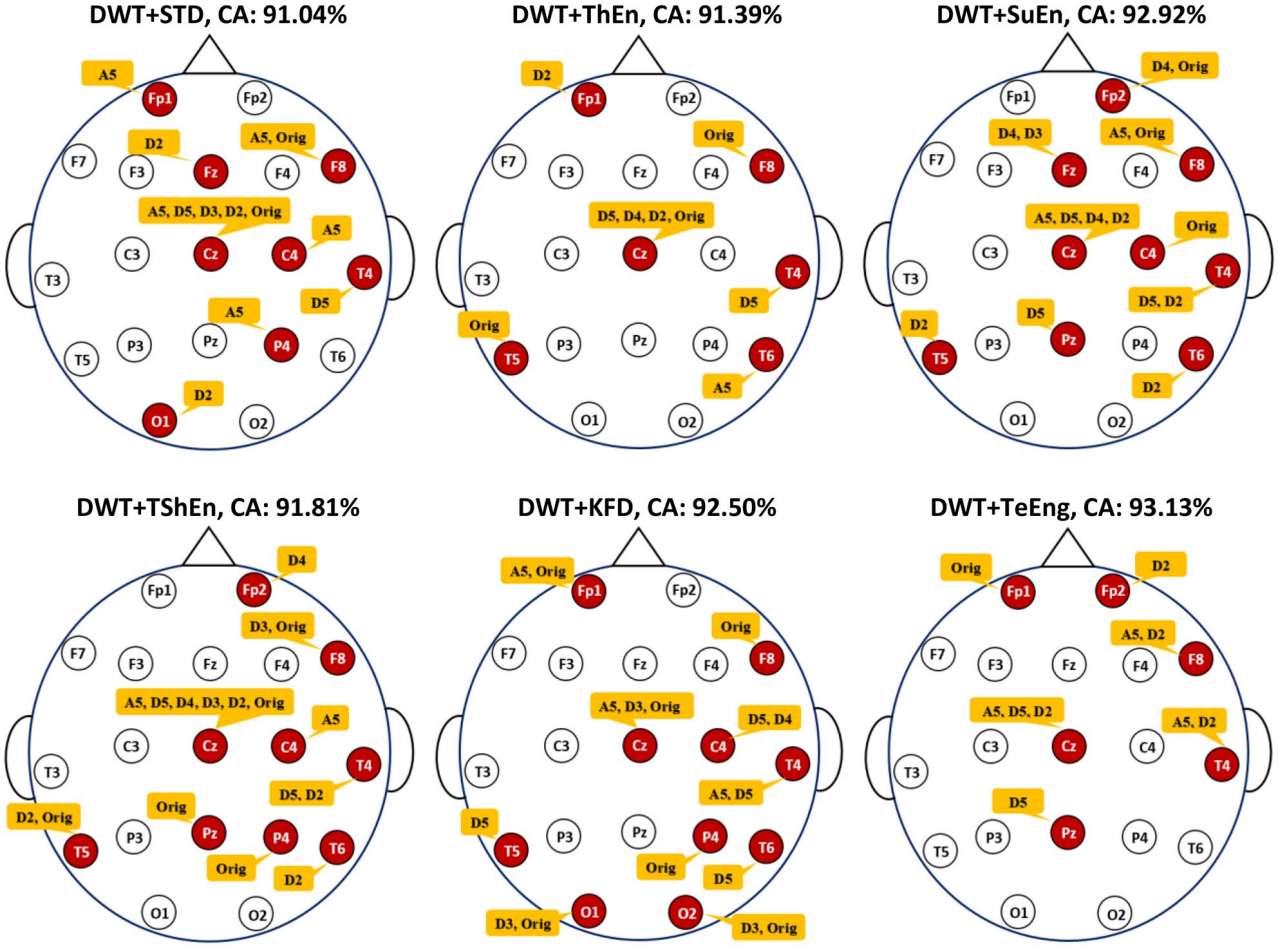
The optimal subsets of features and the channels they belong to when KNN and DWT are used.

In addition, when looking at the results of Experiment III (Tables 6 and 7), the improvement in accuracy scores can also be observed across other combinations. According to^55,56^, certain EEG channels or specific frequency bands may contain more discriminative information related to the task at hand. Therefore, feature selection can improve accuracy by selecting the most relevant features, reducing dimensionality, reducing noise, simplifying the model, and considering task-specific requirements. Figure 9 shows the optimal subset of features for each combination in Table 6, along with the number of channels they are associated with. Several observations can be derived from this figure. First, all solutions are different because of the different measures adopted for feature extraction. It can also be observed that the features contributed by a particular channel could be different from those of other channels. For example, in the VMD-KFD-KNN combination, channel Fp1 contributes three features (Rest, Vmf1, and Orig), channel F8 contributes only one feature (Vmf2), and channel Fp2 does not contribute any features. Furthermore, Fig. 9 highlights that channel Cz has the most significant contribution with the highest number of features, followed by F8. This observation may indicate the importance of these particular channels for MCI versus HC classification. The accuracy improvement can also be observed across the other combinations listed in Table 7. The DWT-TeEng-KNN combination with NSGA-II achieves the highest classification accuracy. With this combination, only ten features selected from six channels lead to an accuracy of 93.13%. Figure 10 shows the optimal subset of features for each combination in Table 7, along with their corresponding channels. The same observations derived from Fig. 9 can also be derived from Fig. 10.

By reviewing the results of the last two experiments (Experiment II and Experiment III), two additional significant observations can also be derived. First, we note that optimizing the features (or subbands) with each individual channel is more effective for improving accuracy than optimizing the channels. Since each channel’s signal is decomposed into sub-signals, each with a different subband, selecting only the useful sub-signals with their corresponding subbands is more effective in improving accuracy. The second observation, which can be noted from Figs. 9 or 10, is that the selected features in a specific channel differ from one measure to another because of the unique information extracted by each measure. Furthermore, in a certain measure, the selected features in Fig. 9 (VMD-based decomposition) differ from those in Fig. 10 (DWT-based decomposition). This is because, as discussed in “Signal decomposition” Section, the resulting subbands produced by VMD differ from those produced by DWT.

When applying other classifiers than KNN, the classification accuracy scores are also improved in both Experiments II and III. Regarding the results of Experiment II listed in Tables 8 and 9, a notable observation is that SVM and DA classifiers outperform RF and KNN across all scenarios. It is worth mentioning that when RF and KNN are employed, they tend to select fewer channels than SVM and DA. By comparing the results of Experiment II and Experiment III, the feature selection results in Tables 10 and 11 (results of Experiment III) show an additional improvement in accuracy when compared to the channel selection results presented in Tables 8 and 9 (results of Experiment II).

**Figure 11:**
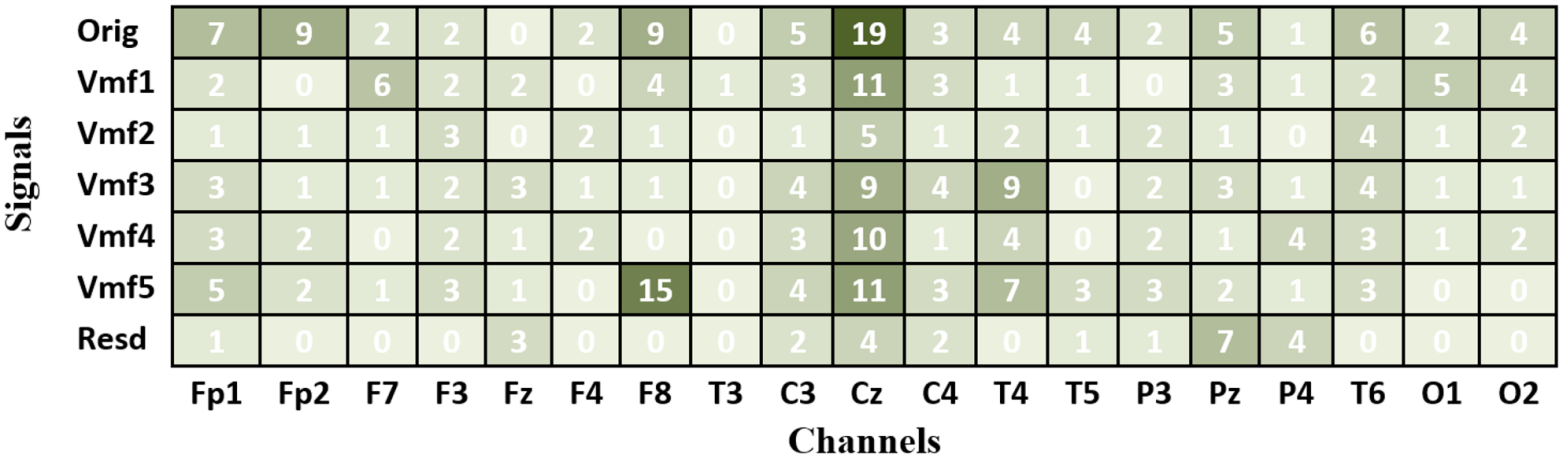
The frequency bands' feature selection counts across the 24 VMD-based combination methods.

**Figure 12:**
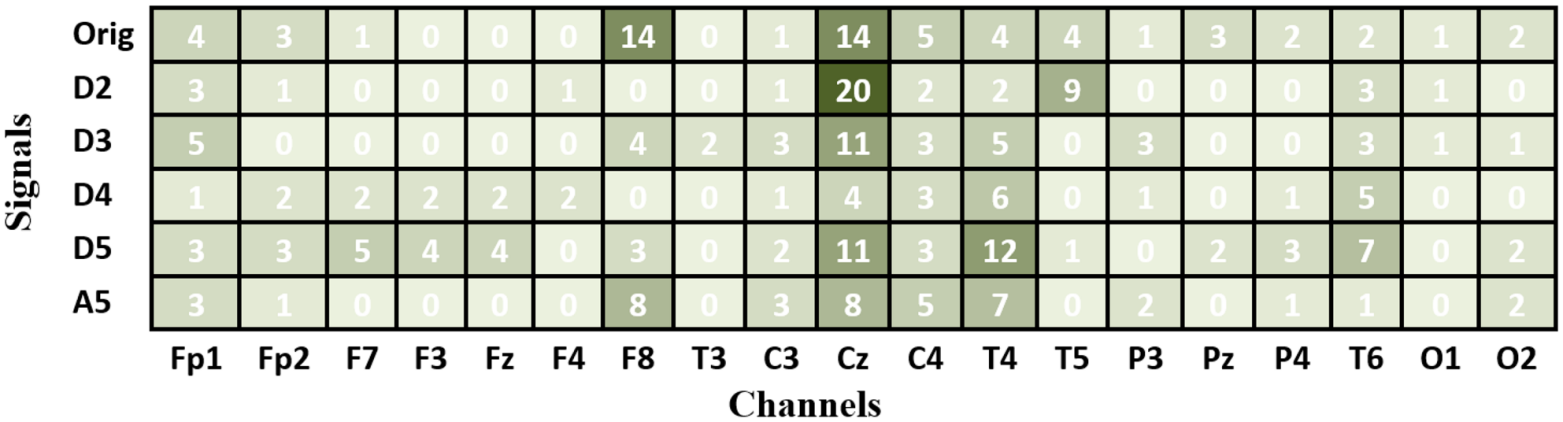
The frequency bands' feature selection counts across the 24 DWT-based combination methods.

Accordingly, the results demonstrate that selecting suitable features is more effective for improving accuracy than optimizing the channels. It is of interest to see which features are most important. To facilitate the discussion, let us recall the feature extraction process described in “Signal decomposition” Section. As shown in Fig. 4, each signal segment within a particular channel is decomposed into sub-signals, each having a unique frequency band. One feature is extracted from each sub-signal or band. An additional feature is computed from the original signal that contains the complete frequency band. To determine the most relevant features, the number of times each feature (in other words, frequency band) is selected in each channel is computed across all the combinations. The results are presented in Fig. 11 for VMD-based combinations and Fig. 12 for DWT-based combinations. In Figs. 11 and 12, each cell has a value representing the number of times a certain feature is chosen from the optimal feature subsets. Several observations are worth discussing in Figs. 11 and 12. First, no specific frequency band is the most selected over all channels, demonstrating that no specific frequency band has absolute dominance across all channels. The figures also show that within a particular channel, some features are more selected than others. In other words, not all features or bands within a specific channel are equally important for extracting features. By focusing on channel columns, the features belonging to channel Cz are the most selected in both figures. This indicates that most of the frequency bands of channel Cz are useful for extracting features but have different levels of importance. Following Cz, channels F8 and T4 also have some bands that are frequently selected. This outcome aligns with the results in Fig. 8, showing that Cz, F8, T4, and Fp1 are the most chosen channels. By looking at the rows of Figs. 11 and 12 (signal bands), several observations can also be derived. The first observation is that features extracted from the original signal segment (Orig) are frequently selected, and in some channels, they are the most frequently selected features. This is primarily because the original segment contains the complete frequency band (0.5–65 Hz) and retains the original information within this band. In addition to the original segment feature, some features from other subbands are also frequently selected within the optimal solutions. This indicates that the information introduced by the complete frequency band is insufficient to achieve the highest accuracy and confirms that more information can be gained from the subbands^34,40^. In other words, hidden patterns at those frequencies can be uncovered by decomposing a signal by VMD or DWT into sub-signals with smaller frequency bands. Since the subbands produced by VMD are different from those produced by DWT, the values in Figs. 11 and 12 differ. The values are also different within a figure from band to band. For example, by looking at Fig. 12, it can be noted that features extracted from the frequency subbands 32–64 Hz (D2), 16–32 Hz (D3), and 4–8 Hz (D5) are frequently selected for channel Cz. In contrast, for channel F8, the features belonging to the original segment and A5 (0.5–4 Hz) are the most frequently selected. For the T4 channel, D5 and A5 features are the most selected.

## Comparison with the literature

Of the 48 combinations (models) built in the present study, the results of eight are summarized in Tables 12 and 13. Table 12 includes the combinations with the corresponding classification accuracy for the three experiments. Compared to the accuracy scores obtained using all channels (Experiment I), NSGA-II succeeded in selecting fewer channels (Experiment II), leading to higher accuracy scores. The accuracy was further improved when a few suitable features were selected (Experiment III). In addition to the classification accuracy obtained based on Experiment III, Table 13 also includes the corresponding sensitivity, specificity, precision, and F-score results. Table 13 shows that the ability of the models to identify healthy people is greater than the ability to identify people with MCI. To compare the current results with those in the literature, three aspects are considered: EEG channel selection, feature selection, and validation methods. Table 14 summarizes the results of methods in the literature that addressed the problem of MCI versus HC classification in the resting state.

**Table 12:** Accuracy improvement in eight combinations when NSGA-II is used for EEG channel and feature selection.

Combination	Classification Accuracy based on:		
	All channels (No_ch)	Subset of channels (No_ch)	Subset of features (No_feat, No_ch)
VMD-KFD-RF	74.24 (19)	85.49 (5)	90.83 (14,10)
VMD-TeEng-SVM	74.24 (19)	91.56 (5)	95.28 (8,7)
VMD-TeEng-DA	79.58 (19)	91.94 (5)	95.63 (15,10)
VMD-SuEn-KNN	62.36 (19)	86.04 (4)	91.53 (10,6)
DWT-ThEn-RF	68.54 (19)	87.64 (3)	90.63 (9,6)
DWT-TeEng-SVM	80.14 (19)	92.64 (5)	95.83 (9,8)
DWT-ThEn-DA	75.42 (19)	94.65 (8)	95.63 (13,8)
DWT-TeEng-KNN	58.54 (19)	85.35 (2)	93.13 (10,6)

**No_ch**: number of selected channels, and **No_feat**: number of selected features.

**Table 13:** Average classification performance (%) in eight combinations with the features selected by NSGA-II.

Combination	Accuracy	Sensitivity	Specificity	Precision	F-score
VMD-KFD-RF	91.83	89.02	92.56	90.47	87.84
VMD-TeEng-SVM	95.28	90.61	99.36	99.17	94.70
VMD-TeEng-DA	95.63	91.10	99.49	99.34	95.02
VMD-SuEn-KNN	91.53	88.94	93.72	92.30	90.59
DWT-ThEn-RF	90.63	88.27	92.30	90.64	89.32
DWT-TeEng-SVM	95.83	90.91	100.0	100.0	95.24
DWT-ThEn-DA	95.63	90.76	99.74	99.67	95.00
DWT-TeEng-KNN	93.13	90.91	95.00	93.90	92.38

**Table 14:** Comparison of our results with those reported in the literature for the MCI versus HC classification.

References	Feature extraction methods	Classification methods	Data used	No. of channels	CA (%)
^24^	Power, relative power, power ratio for different bands	Neurofuzzy + KNN	11 MCI/16 HC	3	88.89% using hold-out validation
^36^	DWT	Decision Tree (C4.5)	Own data,37MCI/ 23 HC	19	93.3% using tenfold CV83.3% using hold out
^25^	Supervised dictionary learning with spectral features, named CLC-KSVD	Same in^24^	3	88.9% using hold-out validation	
^26^	Power spectral features	KNN	Same in^24^	19	81.5%
^27^	SWT + statistical features	SVM	Data from^24^, 11 MCI/ 11 HC	19	96.94% based on intra-subject validation
^28^	Permutation entropy and auto-regressive	ELM	Same in^24^	19	98.78% using tenfold CV
^29^	kernel Eigen-relative-power	SVM	24 MCI/ 27 HC	5	90.2% using LOSO CV
^38^	DWT + PSD + coherence	Bagged Trees	Same in^24^	19	96.5% using fivefold CV
^39^	Power intensity for each high and low-frequency band	KNN	Same in^36^	19	95.0% using tenfold CV
^30^	–	LSTM	Same in^24^	19	96.41% using fivefold CV
^31^	Several features using 10 measures	SVM	Private data,21 MCI/ 21 HC	8	86.85% using LPSO CV
^32^	Spectral, functional connectivity, and nonlinear features	SVM	18 MCI/ 16 HC	19	99.4% using tenfold CV
^33^	DWT leader	AdaBoostM1	Same in^32^	19	93.50% using tenfold CV
^34^	EMD + Log energy entropy	KNN	Data in^24,32^ 29 MCI/ 32 HC	19	97.60% using tenfold CV
^35^	–	CNN	Same in^24^	19	84.28% using LOSO CV
Present study	VMD + TeEng	SVM	Data from^24^, 11 MCI/ 13 HC	7	95.28% and 95.83%, respectively, using LOSO CV and NSGA-II
	DWT + TeEng			8	

In the context of EEG channel or feature selection, only the studies^24,25,27,29,31,32,37^ have explored various strategies to achieve this goal. These strategies include area-based division^24,25^, incremental evaluation^27^, Fisher's class separability criterion^29^, manual symmetric selection^31^, backward-elimination^32^, and the ANOVA test^37^.

Regarding EEG channel selection, there have been attempts in^24,25,27,31^ to decrease the number of channels. In the studies^24,25^, the scalp area was partitioned into five subareas, and classification based on these subareas was performed. In^24^, similar accuracies of 88.89% were obtained for each subarea, with the exception of the frontal subarea, which attained a lower score of accuracy. In^25^, using the same dataset as^24^, the best accuracy, 88.9%, was attained using the left-temporal subarea. In line with^27,31^, the present study aimed to identify the most pertinent channels in various scalp areas. The study^27^ examined channel reduction by employing the incremental evaluation methodology for identifying the optimal subset of channels. The maximum accuracy of 96.94% was attained only in the case of including all 19 channels for the classification. In other words, no channel subset is obtained that achieves higher accuracy than that attained using the entire set of 19 channels. Researchers in^31^ evaluated the classification accuracy by manually selecting channel subsets that are restricted to being symmetric combinations of two, four, six, and eight channels. The highest accuracy of 86.85% was attained when a symmetric combination of eight channels was used. Considering only symmetric channel pairs ignores other channel combinations that may lead to better accuracy scores.

Regarding feature selection, only three studies^29,32,37^ have addressed the problem of decreasing the number of features for MCI vs. HC classification. In^29^, the authors employed Fisher's criterion to identify the most suitable channels and subbands. As a result, the highest accuracy of 90.25% was achieved by seven features extracted from five different channels in various scalp areas (prefrontal, frontal, left, and right temporal). In^32^, 431 feature values were aggregated from all channels using different measures, and a backward-elimination method was employed to select 361 feature values that belong to different channels in various scalp regions, achieving an accuracy of 99.4%. In^37^, the ANOVA test was employed for selecting features, but based on different measures.

Selecting optimal channel or feature combinations is a complex task that necessitates the utilization of effective methods. In our previous study^40^, several methods have also been investigated to select the channels leading to the highest MCI detection accuracy, such as incremental evaluation, backward-elimination, forward-elimination, and heuristic optimization methods. The results demonstrated that the heuristic optimization methods have a greater ability to select a few suitable channels. The limitation of^40^ is that performance validation was performed using k-fold CV, similar to most previous studies in Table 14^27,28,30,32–34,36–38^. As previously discussed, intra-subject validation methods, such as k-fold, do not mimic real-world scenarios and potentially introduce a classification bias. To avoid overestimation of accuracy caused by data leakage, inter-subject validation methods are required. Among the methods in Table 14, the studies^24,25,29,31,36^ have validated their models using different inter-subject validation methods: hold-out^24,25,36^, LPSO^31^, and LOSO^29^. LOSO offers a more comprehensive approach compared to holdout validation and LPSO because it guarantees that all subjects are used for testing in a number of iterations equal to the total number of subjects.

Of all the previous studies, only^29^ used LOSO for validation^29^ also explored selecting the best channels and frequency subbands for identifying MCI. In^29^, EEG segments were partitioned into several subbands, from which features were extracted using four feature extraction methods, each depending on relative power. The outcomes of^29^ demonstrated that for each feature extraction method, different subbands and channels were selected for optimal classification accuracy. For instance, with the first feature extraction method, the highest accuracy was obtained using a single channel (Fp2) with the subbands of beta and gamma, while with the second method, the highest accuracy was attained using five channels: Fp1 (theta and gamma), Fp2 (delta, theta, beta, and gamma), F3 (delta), Fz (beta), and Cp4 (delta). With the other two methods, the relative power features were extracted based on channel pairs (between-electrode relative power). For instance, with the third feature extraction method, the highest accuracy was obtained using the features extracted from Fp2-F7 (delta), Fp2-T3 (gamma), Fp2-T5 (alpha), Fp2-Oz (alpha), Fp2-T6 (alpha), and F7-T6 (alpha). These outcomes demonstrate that no specific frequency band is the most selected over all channels, which we also demonstrated in Figs. 11 and 12. Furthermore, in the present study, based on the 48 combination methods, the results also demonstrated that the selected subbands and channels are different from decomposition method to decomposition method, measure to measure. Figures 9 and 10 include the optimal feature subsets of twelve combination methods when the KNN classifier is used.

Accordingly, the main advantages of the present study can be summarized as follows: Firstly, a more effective EEG channel and feature selection approach is employed compared to the approaches utilized in^24,25,27,29,31,32,37^. Specifically, the study utilizes a heuristic optimization method, NSGA-II, for selecting channels and features, as well as classifier parameters. Furthermore, the study implements the LOSO CV technique for validation purposes, which mimics real-world scenarios and ensures more reliable and unbiased evaluations. Additionally, the study introduces effective and efficient feature extraction methods to develop appropriate biomarkers for detecting MCI. As a result, accurate and practical models are developed with a reduced number of electrodes and features. Moreover, the developed models are evaluated on a publicly available dataset that has also been utilized in^24–28,30,35^. When comparing the outcomes of the present study with those of studies that adopted inter-subject validation methods^24,25,29,31,35^, it is evident that the outcomes of the present study, which employs systematic optimization for channel and feature selection, surpass the outcomes of those studies (refer to Table 14).

Finally, there are issues related to the dataset that need to be highlighted: dataset size, data availability, and unified evaluation. The present study employed a public dataset to evaluate the proposed methods; however, there is still a need to evaluate the methods with a larger dataset. There is a real challenge concerning the availability of MCI datasets. Most of the MCI datasets employed in the literature are either private or small in size. Furthermore, the use of different datasets is a common shortcoming of these types of studies, which renders the comparison of study results unfair. Therefore, it is recommended to establish a standard framework for assessing the researchers' suggested methodologies, which may include the utilization of open-source and large-scale datasets.

## Conclusion and future work

The study focuses on selecting EEG channels and features using a multi-objective optimization method for MCI detection and computing the accuracy using the LOSO CV. The goal is to progress toward building an accurate and practical MCI detection system with a low number of electrodes and features. The study introduces effective and efficient VMD and DWT-based methods for feature extraction. One of these methods is combined with one nonlinear measure. To ensure a comprehensive investigation, various measures and classifiers were employed, resulting in 48 diverse model combinations. Various experiments were conducted to showcase the efficacy of the multi-objective NSGA-II optimization method in the selection of EEG channels and features.

The results demonstrate the effectiveness of the NSGA-II approach in achieving improved classification performance. By using a low number of suitable EEG channels, the LOSO CV-based results showed significant enhancements compared to using all channels. Moreover, the results were further improved by selecting relevant features from different channels. For instance, when using VMD, Teager energy, and SVM, the accuracy increased from 74.24% (using all channels) to 91.56% with only five selected channels. In addition, by selecting eight features from seven channels, the accuracy was further improved to 95.28%. Similarly, in the case of DWT and Teager energy, the accuracy increased from 80.35% (using all channels) to 92.64% with five selected channels. With the selection of nine features from eight channels, the accuracy further improved to 95.83%. These promising results highlight the potential of accurately diagnosing MCI by employing heuristic optimization methods to select a minimal number of suitable electrodes and features.

In this study, only one type of FE measure was used in each model. It would be interesting to study the impact of mixing different types of measures. In this case, heuristic optimization methods can also be used to find the optimal set of features with the objective of improving detection accuracy. To better understand the features chosen by the best accuracy results, an EEG source reconstruction technique can be used after selecting the best channels and features.

## Data Availability

The datasets used are available online at https://misp.mui.ac.ir/en/eeg-data-0.
