# Publisher Correction: Selecting EEG channels and features using multi-objective optimization for accurate MCI detection: validation using leave-one-subject-out strategy

**DOI:** 10.1038/s41598-024-64545-z

**Published:** 2024-06-13

**Authors:** Majid Aljalal, Saeed A. Aldosari, Marta Molinas, Fahd A. Alturki

**Affiliations:** 1 Department of Electrical Engineering, College of Engineering, King Saud University, Riyadh, Saudi Arabia; 2 Department of Engineering Cybernetics, Norwegian University of Science and Technology, Trondheim, Norway

Correction to: *Scientific Reports* 10.1038/s41598-024-63180-y, published online 30 May 2024

The original version of this Article contained errors in Table 2, where the header row stated incorrect items for “VMD” and “DWT”, respectively. The correct and incorrect data appears below.

Incorrect: 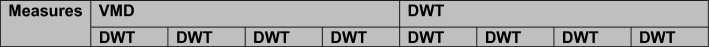


Correct: 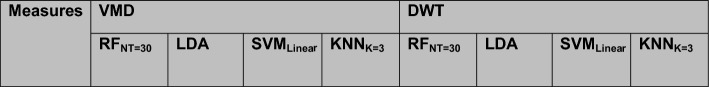


The original Article has been corrected.

